# Genetic polymorphisms of IL-6 promoter in cancer susceptibility and prognosis: a meta-analysis

**DOI:** 10.18632/oncotarget.24033

**Published:** 2018-01-05

**Authors:** Xingchun Peng, Jun Shi, Wanqun Sun, Xuzhi Ruan, Yang Guo, Lunhua Zhao, Jue Wang, Bin Li

**Affiliations:** ^1^ Department of Pathology, Shanghai Xuhui Central Hospital, Zhongshan-Xuhui Hospital, Fudan University, Shanghai Clincal Center, CAS, Shanghai, 200031, P. R. China; ^2^ School of Basic Medical Sciences, Hubei University of Medicine, Shiyan, 442000, Hubei, P. R. China; ^3^ Department of Stomatology, Taihe Hospital, Hubei University of Medicine, Shiyan, 442000, Hubei, P. R. China

**Keywords:** IL-6, cancer, meta-analysis, case-control studies

## Abstract

IL-6 is critical for tumorigenesis. However, previous studies on the association of IL-6 promoter polymorphisms with predisposition to different cancer types are somewhat contradictory. Therefore, we performed this meta-analysis regarding the relationship between IL-6 promoter single nucleotide polymorphisms and cancer susceptibility and prognosis. Up to April 2017, 97 original publications were identified covering three IL-6 promoter SNPs. Our results showed statistically significant association between IL-6 promoter and cancer risk and prognosis. Subgroup analysis indicated that rs1800795 was significantly associated with increased risk of cervical cancer, colorectal cancer, breast cancer, prostate cancer, lung cancer, glioma, non-Hodgkin’s lymphoma and Hodgkin’s lymphoma but not gastric cancer and multiple myeloma. Furthermore, rs1800796 was significantly associated with increased risk of lung cancer, prostate cancer and colorectal cancer but not gastric cancer. Additionally, rs1800797 was significantly association with breast cancer, non-Hodgkin’s lymphoma, B-cell lymphoma and diffuse large B-cell lymphoma but not gastric cancer. Simultaneously, rs1800795 and rs1800796 were associated with a significantly higher risk of cancer in Asia and Caucasian, rs1800797 was associated with a significantly risk of cancer in Caucasian but not in Asia. Furthermore, IL-6 promoter polymorphisms were significantly associated with the prognosis of cancer. Considering these promising results, IL-6 promoter including rs1800795, rs1800796 and rs1800797 may be a tumor marker for cancer therapy.

## INTRODUCTION

Interleukin-6 (IL-6) is one of the most widely recognized cytokines. It can regulate immune responses and cell proliferation and differentiation [1]. IL-6 was originally studied as an inflammatory factor, which was later found to be closely related to tumorigenesis, invasion and metastasis [2]. High expression of IL-6 is associated with different cancer types, such as esophageal cancer, non-small cell lung cancer, endometrial cancer, breast cancer, prostate cancer, lung cancer, chronic lymphocytic leukemia and diffuse large B-cell lymphoma [2–5]. Therefore, IL-6 is closely related to tumor occurrence and development, and understanding the genetic diversity of IL-6 will be helpful for cancer risk prediction and gene therapy.

The human IL-6 gene is located on chromosome 7p21 which is identified as pro-inflammatory cytokine [6], and plays an important role in the pathogenesis of several types of cancers. The single nucleotide polymorphisms (SNPs) at the 50 flanking region of the IL-6 gene promoter (rs1800795, rs1800796 and rs1800797) can effect on IL-6 expression [7–9]. However, previous studies have conflicting results between IL-6 promoter (rs1800795, rs1800796 and rs1800797) and cancer susceptibility [10–99] and prognosis [40, 47, 53, 57, 63, 100-106].

To confirm whether IL-6 promoter polymorphisms are related to cancer risk, we performed this meta-analysis, aiming to measure the correlation between IL-6 promoter polymorphisms and cancer susceptibility and prognosis.

## RESULTS

### Characteristics of published studies

**Figure 1:**
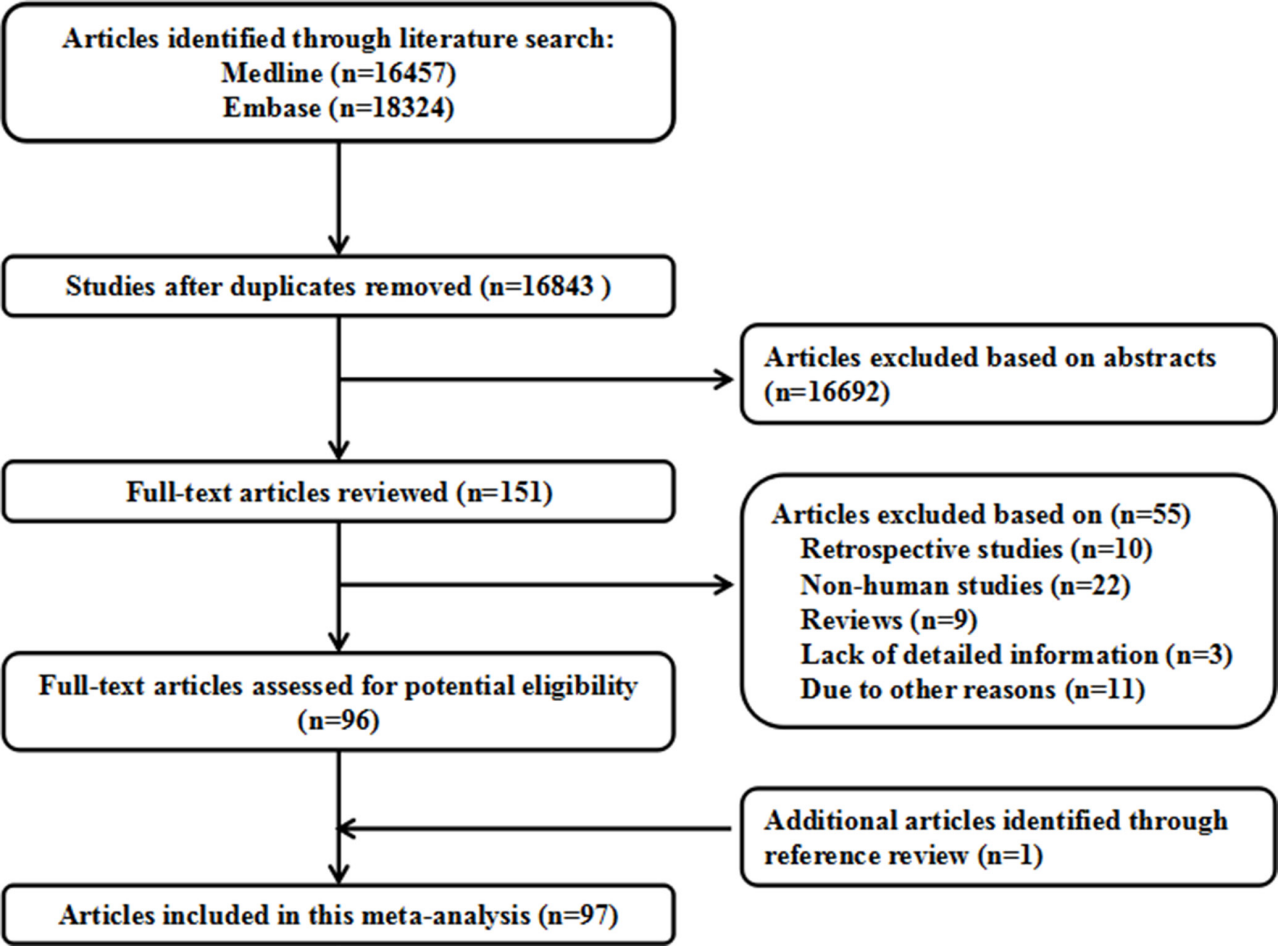
Flow diagram of the study selection process

A flow chart was carefully identified of the search process in Figure 1. After duplicates removed, 16843 studies were retrieved (PubMed: 16457, Embase: 18324). Finally, ninety-seven studies were chosen, and the data was extracted. Seventy-eight studies reported the association between rs1800795 and cancer risk, twenty-one studies reported the association between rs1800796 and cancer risk, seventeen studies reported the association between rs1800797 and cancer risk, and twelve studies reported the association between IL-6 promoter polymorphisms and cancer prognosis. The genotype frequencies of IL-6 promoter in controls of each study met the HWE expectation (*P* > 0.05). The genotype distributions of all studies are summarized in Supplementary Tables 1–6.

### Meta-analysis of rs1800795 polymorphism and cancer risk

Seventy-eight studies reported the association between rs1800795 and cancer risk. Our results showed that rs1800795 was significantly associated with increased cancer risk in allelic, dominant, recessive and additive models (OR = 1.05, 95% CI: 1.01, 1.09, *P* = 0.007, allelic models respectively) (Table 1). Subgroup analysis indicated that rs1800795 was associated with a significantly higher risk of cancer in Asia (OR = 1.05, 95% CI: 1.01, 1.10, *P* = 0.003, allelic models respectively) (Table 2) and Caucasian (OR= 1.04, 95% CI: 1.02, 1.06, *P* < 0.001, allelic models respectively) (Table 2) in all four gene model. Meanwhile, rs1800795 was significantly associated with increased risk of cervical cancer (OR = 1.13, 95% CI: 1.05, 1.21, *P* = 0.004, allelic models respectively) (Table 3), colorectal cancer (OR = 1.10, 95% CI: 1.02, 1.19, *P* = 0.014, allelic models respectively) (Table 3), breast cancer (OR = 1.08, 95% CI: 1.01, 1.19, *P* = 0.013, allelic models respectively) (Table 3), prostate cancer (OR = 1.08, 95% CI: 1.03, 1.13, *P* = 0.005, allelic models respectively) (Table 3), lung cancer(OR = 1.08, 95% CI: 1.02, 1.15, *P* = 0.003, allelic models respectively) (Table 3), glioma (OR = 1.28, 95% CI: 1.13, 1.46, *P* < 0.001, allelic models respectively) (Table 3), non-hodgkin’s lymphoma (OR = 1.25, 95% CI: 1.01, 1.51, *P* = 0.049, allelic models respectively) (Table 3) and hodgkin’s lymphoma (OR = 1.22, 95% CI: 1.02, 1.45, *P* = 0.030, allelic models respectively) (Table 3) but not gastric cancer (OR = 0.95, 95% CI: 0.83, 1.08, *P* = 0.435, allelic models respectively)(Table 3) and multiple myeloma (OR = 1.06, 95% CI: 0.88, 1.30, *P* = 0.559, allelic models respectively) (Table 3) in all four gene model.

**Table 1:** Meta-analysis of IL-6 promoter polymorphisms and cancer susceptibility

Genetic model	No.of studies	Heterogeneity		OR	95% CI	*P* value	Model
		I^2^	*P* value				
**rs1800795**	**78 (46096/56969)**						
G vs. C		55.5%	0.000	1.05	1.01,1.09	0.007	Random-effects model
GG+ GC vs. CC		38.4%	0.000	1.04	1.01,1.08	0.021	Fixed-effects model
GG vs. GC+CC		55.1%	0.000	1.08	1.03,1.13	0.001	Random-effects model
GG vs. GC		53.4%	0.000	1.06	1.00,1.14	0.035	Random-effects model
**rs1800796**	**21 (9930/13080)**						
C vs. G		47.2%	0.008	1.11	1.04,1.18	< 0.001	Fixed-effects model
CC+ CG vs. GG		34.4%	0.059	1.12	1.02,1.21	0.029	Fixed-effects model
CC vs. CG+GG		50.6%	0.003	1.09	1.03,1.16	0.045	Random-effects model
CC vs. CG		44.5%	0.013	1.04	1.01,1.09	0.010	Fixed-effects model
**rs1800797**	**17 (9162/12724)**						
G vs. A		0.0%	0.901	1.04	1.01,1.08	0.007	Fixed-effects model
GG+ GA vs. AA		0.0%	0.904	1.07	1.02,1.13	0.007	Fixed-effects model
GG vs. GA+AA		0.0%	0.493	1.06	1.03,1.09	0.004	Fixed-effects model
GG vs. GA		38.0%	0.020	1.03	1.00,1.08	0.035	Fixed-effects model

**Table 2:** Meta-analysis of IL-6 promoter polymorphisms and cancer risk in ethnicity

rs1800795	No.of studies	Heterogeneity		OR	95% CI	*P* value	Model
Ethnicity		I^2^	*P* value				
**Asian**	**3 (1090/1482)**						
G vs. C		75.5%	0.017	1.05	1.01,1.10	0.003	Random-effects model
GG+ GC vs. CC		47.1%	0.151	1.03	1.01,1.06	< 0.001	Fixed-effects model
GG vs. GC+CC		66.8%	0.049	1.07	1.03,1.12	< 0.001	Random-effects model
GG vs. GC		56.7%	0.100	1.06	1.01,1.12	0.002	Random-effects model
**Caucasian**	75 (44895/55402)						
G vs. C		49.7%	0.000	1.04	1.02,1.06	< 0.001	Fixed-effects model
GG+ GC vs. CC		34.0%	0.001	1.05	1.02,1.09	0.004	Fixed-effects model
GG vs. GC+CC		51.4%	0.000	1.10	1.06,1.15	< 0.001	Random-effects model
GG vs. GC		51.6%	0.000	1.08	1.03,1.14	0.004	Random-effects model
**rs1800796**							
**Asian**	**12 (3574/4423)**						
C vs. G		52.9%	0.013	1.08	1.03,1.14	< 0.001	Random-effects model
CC+ CG vs. GG		28.3%	0.160	1.12	1.05,1.20	< 0.001	Fixed-effects model
CC vs. CG+GG		54.9%	0.009	1.15	1.06,1.25	0.009	Random-effects model
CC vs. CG		53.2%	0.012	1.06	1.02,1.11	0.018	Random-effects model
**Caucasian**	**9 (5679/8001)**						
C vs. G		0.0%	0.651	1.05	1.01,1.10	0.003	Fixed-effects model
CC+ CG vs. GG		5.2%	0.392	1.05	1.02,1.09	< 0.001	Fixed-effects model
CC vs. CG+GG		15.2%	0.299	1.06	1.02,1.11	0.002	Fixed-effects model
CC vs. CG		29.9%	0.180	1.04	1.02,1.06	< 0.001	Fixed-effects model
**rs1800797**							
**Asian**	**2 (187/495)**						
G vs. A		0.0%	0.637	1.23	0.79,2.04	0.326	Fixed-effects model
GG+ GA vs. AA		0.0%	0.954	4.38	1.21,15.9	0.025	Fixed-effects model
GG vs. GA+AA		0.0%	0.589	0.86	0.48,1.34	0.405	Fixed-effects model
GG vs. GA		0.0%	0.377	0.38	0.21,0.68	0.001	Fixed-effects model
**Caucasian**	**14 (8298/11573)**						
G vs. A		0.0%	0.806	1.04	1.01,1.08	0.041	Fixed-effects model
GG+ GA vs. AA		0.0%	0.900	1.06	1.02,1.11	0.034	Fixed-effects model
GG vs. GA+AA		3.1%	0.418	1.06	1.01,1.11	0.014	Fixed-effects model
GG vs. GA		25.9%	0.122	1.03	1.01,1.06	0.002	Fixed-effects model

**Table 3:** Subground of analyses of rs1800795 polymorphism and cancer risk

rs1800795	No.of studies	Heterogeneity		OR	95% CI	*P* value	Model	
Cancer type		I^2^	*P* value					
**Cervical cancer**	**7 (1734/2272)**							
G vs. C		70.1%	0.003	1.13	1.05,1.21	0.004	Random-effects model	
GG+ GC vs. CC		58.5%	0.025	1.16	1.06,1.27	0.039	Random-effects model	
GG vs. GC+CC		58.7%	0.024	1.21	1.08,1.34	0.002	Random-effects model	
GG vs. GC		44.0%	0.098	1.19	1.08,1.30	0.003	Fixed-effects model	
**Colorectal cancer**	**14 (7399/9808)**							
G vs. C		63.7%	0.000	1.10	1.02,1.19	0.014	Random-effects model	
GG+ GC vs. CC		0.0%	0.515	1.13	1.04,1.22	0.003	Fixed-effects model	
GG vs. GC+CC		63.6%	0.000	1.11	1.01,1.22	0.047	Random-effects model	
GG vs. GC		54.6%	0.006	1.07	1.01,1.19	0.019	Random-effects model	
**Gastric cancer**	**4 (672/1614)**							
G vs. C		0.0%	0.776	0.95	0.83,1.08	0.435	Fixed-effects model	
GG+ GC vs. CC		0.0%	0.573	1.03	0.81,1.32	0.799	Fixed-effects model	
GG vs. GC+CC		0.0%	0.874	0.87	0.72,1.06	0.181	Fixed-effects model	
GG vs. GC		0.0%	0.409	0.85	0.69,1.05	0.122	Fixed-effects model	
**Breast cancer**	**13 (9532/15064)**							
G vs. C		57.8%	0.011	1.08	1.01,1.19	0.013		Random-effects model
GG+ GC vs. CC		61.6%	0.004	1.19	1.04,1.34	0.011		Random-effects model
GG vs. GC+CC		60.5%	0.005	1.20	1.06,1.25	0.028		Random-effects model
GG vs. GC		57.2%	0.012	1.11	1.06,1.17	0.009		Random-effects model
**Prostate cancer**	**5 (12169/13116)**							
G vs. C		31.0%	0.203	1.08	1.03,1.13	0.005		Fixed-effects model
GG+ GC vs. CC		32.4%	0.193	1.11	1.04,1.18	0.003		Fixed-effects model
GG vs. GC+CC		24.0%	0.254	1.13	1.05,1.22	0.008		Fixed-effects model
GG vs. GC		15.4%	0.315	1.07	1.02,1.12	0.003		Fixed-effects model
**Lung Cancer**	**4 (3203/3332)**							
G vs. C		0.0%	0.817	1.08	1.02,1.15	0.003		Fixed-effects model
GG+ GC vs. CC		0.0%	0.912	1.06	1.01,1.11	0.002		Fixed-effects model
GG vs. GC+CC		25.4%	0.258	1.07	1.03,1.12	<0.001		Fixed-effects model
GG vs. GC		0.0%	0.745	1.10	1.03,1.17	0.003		Fixed-effects model
**Glioma**	**3 (1082/1701)**							
G vs. C		0.0%	0.482	1.28	1.13,1.46	< 0.001		Fixed-effects model
GG+ GC vs. CC		67.6%	0.046	1.15	1.05,1.26	0.021		Random-effects model
GG vs. GC+CC		77.9%	0.011	1.50	1.03,2.17	0.035		Random-effects model
GG vs. GC		88.7%	0.000	1.55	1.05,2.72	0.012		Random-effects model
**Multiple myeloma**	**5 (6013/6471)**							
G vs. C		0.0%	0.901	1.06	0.88,1.30	0.559		Fixed-effects model
GG+ GC vs. CC		0.0%	0.987	1.00	0.66,1.53	0.992		Fixed-effects model
GG vs. GC+CC		0.0%	0.617	0.95	0.70,1.28	0.733		Fixed-effects model
GG vs. GC		0.0%	0.737	1.01	0.73,1.38	0.961		Fixed-effects model
**Non-Hodgkin’s lymphoma**	**4 (5609/5649)**							
G vs. C		60.9%	0.053	1.25	1.01,1.51	0.049		Random-effects model
GG+ GC vs. CC		11.2%	0.337	1.26	1.03,1.54	0.022		Fixed-effects model
GG vs. GC+CC		50.3%	0.110	1.20	1.04,1.40	0.015		Random-effects model
GG vs. GC		20.1%	0.289	1.15	1.08,1.35	0.008		Fixed-effects model
**Hodgkin’s lymphoma**	**3 (533/484)**							
G vs. C		16.7%	0.301	1.22	1.02,1.45	0.030		Fixed-effects model
GG+ GC vs. CC		0.0%	0.460	1.25	1.02,1.73	0.043		Fixed-effects model
GG vs. GC+CC		0.0%	0.434	1.32	1.02,1.73	0.037		Fixed-effects model
GG vs. GC		0.0%	0.601	1.28	1.08,1.68	0.013		Fixed-effects model

### Meta-analysis of rs1800796 polymorphism and cancer risk

Twenty-one studies reported the association between rs1800796 and cancer risk. Our results showed that rs1800796 was significantly associated with increased cancer risk in allelic, dominant, recessive, and additive models (OR = 1.11, 95% CI: 1.04, 1.18, *P* < 0.001, allelic models respectively) (Table 1). Subgroup analysis indicated that rs1800796 was significantly associated with increased risk of lung cancer (OR = 1.23, 95% CI: 1.11, 1.36, *P* = 0.002, allelic models respectively) (Table 4), prostate cancer (OR = 1.13, 95% CI: 1.04, 1.23, *P* = 0.002, allelic models respectively) (Table 4) and colorectal cancer (OR = 1.07, 95% CI: 1.04, 1.23, *P* < 0.001, allelic models respectively) (Table 4) but not gastric cancer (OR = 1.03, 95% CI: 0.82, 1.29, *P* = 0.786, allelic models respectively) (Table 4) in all four gene model. Furthermore, rs1800796 was associated with a significantly risk of cancer in Asia (OR = 1.08, 95% CI: 1.03, 1.14, *P* < 0.001, allelic models respectively) (Table 2) and Caucasian (OR = 1.05, 95% CI: 1.01, 1.10, P-0.003, allelic models respectively) (Table 2) in all four gene model.

**Table 4:** Subground of analyses of rs1800796 polymorphism and cancer risk

rs1800796	No.of studies	Heterogeneity		OR	95% CI	*P* value	Model
Cancer type		I^2^	*P* value				
**Lung cancer**	**6 (1974/2879)**						
C vs. G		55.6%	0.046	1.23	1.11,1.36	0.002	Random-effects model
CC+ CG vs. GG		57.4%	0.039	1.17	1.09,1.26	0.012	Random-effects model
CC vs. CG+GG		62.0%	0.022	1.15	1.05,1.26	0.012	Random-effects model
CC vs. CG		66.2%	0.011	1.18	1.11,1.27	0.008	Random-effects model
**Prostate cancer**	**5 (2360/3872)**						
C vs. G		0.0%	0.803	1.13	1.04,1.23	0.002	Fixed-effects model
CC+ CG vs. GG		0.0%	0.623	1.18	1.09,1.25	0.018	Fixed-effects model
CC vs. CG+GG		0.0%	0.493	1.19	1.07,1.32	0.015	Fixed-effects model
CC vs. CG		13.5%	0.328	1.16	1.06,1.28	0.014	Fixed-effects model
**Colorectal cancer**	**2 (2581/3363)**						
C vs. G		0.0%	0.826	1.07	1.03,1.12	< 0.001	Fixed-effects model
CC+ CG vs. GG		0.0%	0.859	1.08	1.02,1.15	< 0.001	Fixed-effects model
CC vs. CG+GG		0.0%	0.865	1.10	1.02,1.19	0.006	Fixed-effects model
CC vs. CG		0.0%	0.905	1.15	1.04,1.27	0.009	Fixed-effects model
**Gastric cancer**	**2 (365/395)**						
C vs. G		0.0%	0.910	1.03	0.82,1.29	0.786	Fixed-effects model
CC+ CG vs. GG		0.0%	0.380	1.05	0.62,1.80	0.848	Fixed-effects model
CC vs. CG+GG		0.0%	0.602	1.04	0.78,1.38	0.807	Fixed-effects model
CC vs. CG		26.6%	0.256	1.05	0.78,1.41	0.757	Fixed-effects model

### Meta-analysis of rs1800797 polymorphism and cancer risk

Seventeen studies reported the association between rs1800797 and cancer risk. Our results showed that rs1800797 was significantly associated with increased cancer risk in allelic, dominant, recessive, and additive models (OR = 1.04, 95% CI: 1.01, 1.08, *P* = 0.002, allelic models respectively) (Table 1). Subgroup analysis indicated that rs1800797 has significant association in breast cancer (OR = 1.14, 95% CI: 1.06, 1.23, *P* = 0.002, allelic models respectively) (Table 5), non-Hodgkin’s lymphoma (OR = 1.09, 95% CI: 1.03, 1.05, *P* = 0.006, allelic models respectively) (Table 5), B-NHL (OR= 1.10, 95% CI: 1.03, 1.18, *P* = 0.006, allelic models respectively) (Table 5) and DLCBL (OR = 1.10, 95% CI: 1.01, 1.20, *P* = 0.006, allelic models respectively) (Table 5) but not gastric cancer (OR = 1.04, 95% CI: 0.93, 1.15, *P* = 0.530, allelic models respectively) (Table 5) in all four gene model. Besides, rs1800797 was associated with a significantly higher risk of cancer in Caucasian (OR= 1.04, 95% CI: 1.01, 1.08, *P* = 0.041, allelic models respectively) (Table 2) but not in Asia (OR = 1.23, 95% CI: 0.79, 2.04, *P* = 0.326, allelic models respectively) (Table 2) in all four gene model.

**Table 5:** Subground of analyses of rs1800797 polymorphism and cancer risk

rs1800797	No.of studies	Heterogeneity			OR	95% CI	*P* value	Model
Cancer type		I^2^	*P* value					
**Breast Cancer**	**2 (1164/1388)**							
G vs. A		0.0%		0.705	1.14	1.06,1.23	0.002	Fixed-effects model
GG+ GA vs. AA		0.0%		0.923	1.09	1.02,1.16	< 0.001	Fixed-effects model
GG vs. GA+AA		0.0%		0.454	1.17	1.09,1.15	0.003	Fixed-effects model
GG vs. GA		0.0%		0.365	1.06	1.02,1.11	0.003	Fixed-effects model
**Gastric cancer**	**2 (286/316)**							
G vs. A		0.0%		0.879	1.04	0.93,1.15	0.530	Fixed-effects model
GG+ GA vs. AA		0.0%		0.692	1.01	0.82,1.24	0.936	Fixed-effects model
GG vs. GA+AA		0.0%		0.662	1.06	0.92,1.23	0.429	Fixed-effects model
GG vs. GA		4.0%		0.353	0.99	0.72,1.35	0.934	Fixed-effects model
**Non-Hodgkin’s lymphoma**	**4 (5729/6036)**							
G vs. A		0.0%		0.554	1.09	1.03,1.15	0.006	Fixed-effects model
GG+ GA vs. AA		0.0%		0.497	1.07	1.02,1.13	0.002	Fixed-effects model
GG vs. GA+AA		32.2%		0.219	1.12	1.04,1.21	0.008	Fixed-effects model
GG vs. GA		67.6%		0.026	1.19	1.06,1.32	0.015	Random-effects model
**B-cell lymphoma**	**3 (2161/2018)**							
G vs. A		0.0%		0.736	1.10	1.03,1.18	0.006	Fixed-effects model
GG+ GA vs. AA		0.0%		0.389	0.83	0.50,1.37	0.462	Fixed-effects model
GG vs. GA+AA		0.0%		0.603	1.39	1.12,1.67	0.007	Fixed-effects model
GG vs. GA		58.3%		0.091	1.52	1.21,1.84	0.018	Random-effects model
**DLCBL**	**4 (5388/7026)**							
G vs. A		6.3%		0.344	1.10	1.01,1.20	0.006	Fixed-effects model
GG+ GA vs. AA		0.0%		0.759	1.06	1.01,1.12	< 0.001	Fixed-effects model
GG vs. GA+AA		0.0%		0.683	1.13	1.03,1.24	0.003	Fixed-effects model
GG vs. GA		0.0%		0.830	1.16	1.05,1.28	0.006	Fixed-effects model

DLCBL: diffuse large B-cell lymphoma.

### Meta-analysis of IL-6 promoter polymorphisms and cancer prognosis

Twelve studies reported the association between IL-6 promoter polymorphisms and cancer prognosis. Prognostic meta-analyses were performed in a double gene model: CC vs. GC+GG and GG vs. GC+CC in rs1800795, GG vs. GC+CC in rs1800796 and GG vs. GA+AA in rs1800797. Our results showed that rs1800795, rs1800796 and rs1800797 were significantly associated with cancer prognosis (Table 6).

**Table 6:** Meta-analysis of IL-6 promoter polymorphisms and cancer prognosis

Genetic model	No.of studies	Heterogeneity			HR	95% CI	*P* value	Model
		I^2^	*P* value					
**rs1800795**	**10 (7640/8361)**							
GG vs. GC+CC		0.088		43.6%	1.17	1.07,1.36	< 0.001	Fixed-effects model
CC VS. GC+GG		0.610		0.0%	1.51	1.09,2.13	< 0.001	Fixed-effects model
**rs1800796**	**2 (452/538)**							
GG vs. GC+CC		0.326		0.0%	1.16	1.07,2.42	< 0.001	Fixed-effects model
**rs1800797**	**3 (892/951)**							
GG vs. GA+AA		0.416		0.0%	1.23	1.11,1.37	< 0.001	Fixed-effects model

### Sensitivity analysis

Sensitivity analysis was conducted to assess the stability of the results. The results show four genetic model were stable in Supplementary Figures 1–3.

### Publication bias

Each study in this meta-analysis was performed to evaluate the publication bias by both Begg’s funnel plot and Egger’s test. The results show no obvious evidence of publication bias was found in allelic, dominant, recessive or additive genetic model in Table 7.

**Table 7:** Publication bias analysis of the meta-analysis

Genetic model	Test	t	95% CI	*P*
**rs1800795**				
G vs. C	Begg’s test			0.853
	Egger’s test	-1.49	-3.43,0.48	0.139
GG+ GC vs. CC	Begg’s test			0.272
	Egger’s test	-4.09	-0.84,-027	0.125
GG vs. GC+CC	Begg’s test			0.472
	Egger’s test	-3.27	-5.21,1.11	0.086
GG vs. GC	Begg’s test			0.791
	Egger’s test	-1.74	-0.48,6.99	0.403
**rs1800796**				
C vs. G	Begg’s test			0.602
	Egger’s test	-4.82	-2.60,1.17	0.130
CC+ CG vs. GG	Begg’s test			0.117
	Egger’s test	-9.04	-0.09,0.02	0.070
CC vs. CG+GG	Begg’s test			0.602
	Egger’s test	-5.03	-3.15,1.36	0.125
CC vs. CG	Begg’s test			0.602
	Egger’s test	-5.22	-2.82,1.17	0.121
**rs1800797**				
G vs. A	Begg’s test			0.713
	Egger’s test	-1.23	-8.24,2.07	0.230
GG+ GA vs. AA	Begg’s test			0.890
	Egger’s test	-1.29	-0.87,0.20	0.211
GG vs. GA+AA	Begg’s test			0.931
	Egger’s test	-0.86	-17.0,6.89	0.395
GG vs. GA	Begg’s test			0.973
	Egger’s test	2.28	0.73,14.4	0.531

## DISCUSSION

Cancer is now a public health crisis, affecting millions of people in both developed and developing countries. By 2020, the disease is forecasted to be the major cause of morbidity and mortality in most developing nations [107]. To improve this embarrassing situation, risk factors concerning cancer should be identified timely and controlled effectively. The etiology of cancer involves both genetic and environmental factors. Therefore, understanding the impact of genetic factors on cancer will help to prevent cancer. IL-6 is a confirmed pleiotropic pro-inflammatory cytokine associated with cardiovascular diseases. Elevated expression of IL-6 and its major effector have been implicated in the different stages of cancer development, including initiation, promotion, malignant conversion, invasion, and metastasis [2].

Several recent meta-analysis have focused on the association between IL-6 promoter polymorphisms and cancer risk. Two meta-analysis showed that rs1800795 polymorphism increased the risk of prostate cancer and cervical cancer [108, 109]. Though, the result same with ours, it still exist some problems. On the one hand, single case-control studies with small sample sizes may have weak statistical power, thereby interfering with the precision of their results. On the another hand, the quantity of SNPs involving in their meta-analysis was smaller, which weak the persuasive power of their research. Additionally, no meta-analyses concerning the relationship between IL-6 promoter polymorphisms and cancer prognosis.

In this current meta-analysis was based on 97 case-control study, with 80361 cases and 78712 control from sixteen countries, thus, this meta-analysis provides the most up-to-date epidemiological evidence supporting IL-6 promoter polymorphisms were significantly associated with the susceptibility and prognosis of cancer. To our knowledge, this is the first complete study to identify the potential association between IL-6 promoter and cancer risk and prognosis. However, we also found rs1800795 was not associated with gastric cancer and multiple myeloma, this may be due to tumor heterogeneity or insufficient statistical power to check an association. therefore, a greater number of original case-control studies must be performed to further evaluate the association between the IL-6 promoter polymorphisms and different cancer types.

Although, we performed this meta-analysis very carefully, however, some limitations must be considered in the current meta-analysis. Firstly, we performed stratification only by ethnicity and cancer type, without referring other factors. Further research should be conducted in different sex of population. Secondly, we only select literature that written by English, other language should be chosen in the further. Thirdly, a lack of original data limited further evaluations of the potential gene-gene and gene-environment interactions.

In conclusion, our findings underscore the notion that IL-6 promoter polymorphisms were significantly associated with the susceptibility and prognosis of cancer. In the future, large-scale case-control and population based association studies must be performed in the future to validate the risk identified in the current meta-analysis, and investigate the effect of potential gene-gene and gene-environment interactions on cancer risk.

## MATERIALS AND METHODS

### Search strategy and selection criteria

The selection process is shown in the flow chart (Figure 1). We searched PubMed and Embase databases up to April, 2017, with keywords including “IL-6” or “interleukin-6” and “single nucleotide polymorphism” or “SNP” and “cancer” or “tumor”. Eligible studies were choosing and other relevant publications were also examined.

### Data extraction

The following information in studies were investigated by two independent researchers: (1) first author; (2) publication year; (3) country; (4) cancer type; (5) cases and controls sample size; (6) genotype.

### Statistical analysis

STATA software 12.0 (STATA Corp, College Station, TX, USA) was used to evaluate the relationships between IL-6 promoter polymorphisms and cancer risk and prognosis. Studies were assessed by chi-square in control group under Hardy-Weinberg equilibrium (HWE) to calculate frequencies of IL-6 promoter, and if *P* < 0.05, study was considered to be disequilibrium. The strength of the relationship between IL-6 promoter polymorphisms and the risk of cancer were evaluated by odd ratios with corresponding 95% confidence intervals. The correlation between IL-6 promoter polymorphisms and prognosis of cancer were measured by hazard ratios (HRs). By using Q test and I^2^ statistic to assess heterogeneity among studies in rs1800795 in the allelic (G vs. C), dominant (GG+ GC vs. CC), recessive (GG vs. GC+CC) and additive (GG vs. GC), in rs1800796 in the allelic (C vs. G), dominant (CC+CG vs. GG), recessive (CC vs. CC+GG), and additive (CC vs. CG) genetic models and in rs1800797 in the allelic (G vs. A), dominant (GG+GA vs. AA), recessive (GG vs.GA+AA, and additive (GG vs. GA) genetic models. Random-effect model was chosen if *P*_Q_<0.10 or I^2^ >50%, otherwise, fixed-effect mode was applied. Sensitivity analysis was conducted to assess the stability of the results. Begg’s and Egger’s tests were used to assess the publication bias of each study.

## SUPPLEMENTARY MATERIALS FIGURES AND TABLES








