# Supplementary material for: Genetic polymorphisms of IL-6 promoter in cancer susceptibility and prognosis: a meta-analysis
Source: Oncotarget. 2018 Jan 5;9(15):12351–64. doi: 10.18632/oncotarget.24033 (PMC5844752; doi:10.18632/oncotarget.24033)
Supplement: Supplementary file 1 [file oncotarget-09-12351-s001.pdf]

Genetic polymorphisms of IL-6 promoter polymorphisms in cancer susceptibility and prognosis: a meta-analysis

SUPPLEMENTARY MATERIALS

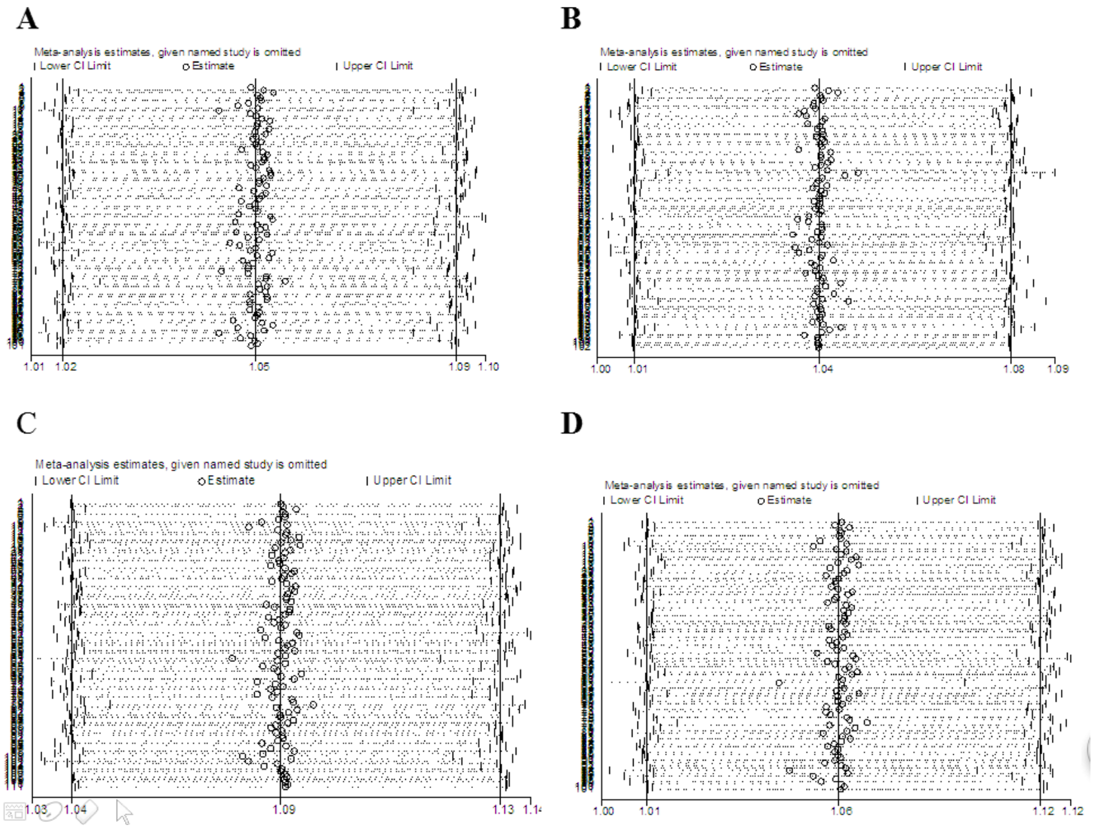

Supplementary Figure 1: Forest plots of sensitivity analysis for the meta-analysisn of rs1800795 and risk of cancer. (**A**) allelic (G vs. C). (**B**) Dominant (GG+ GC vs. CC). (**C**) Recessive (GG vs. GC+CC). (**D**) Additive (GG vs. GC).

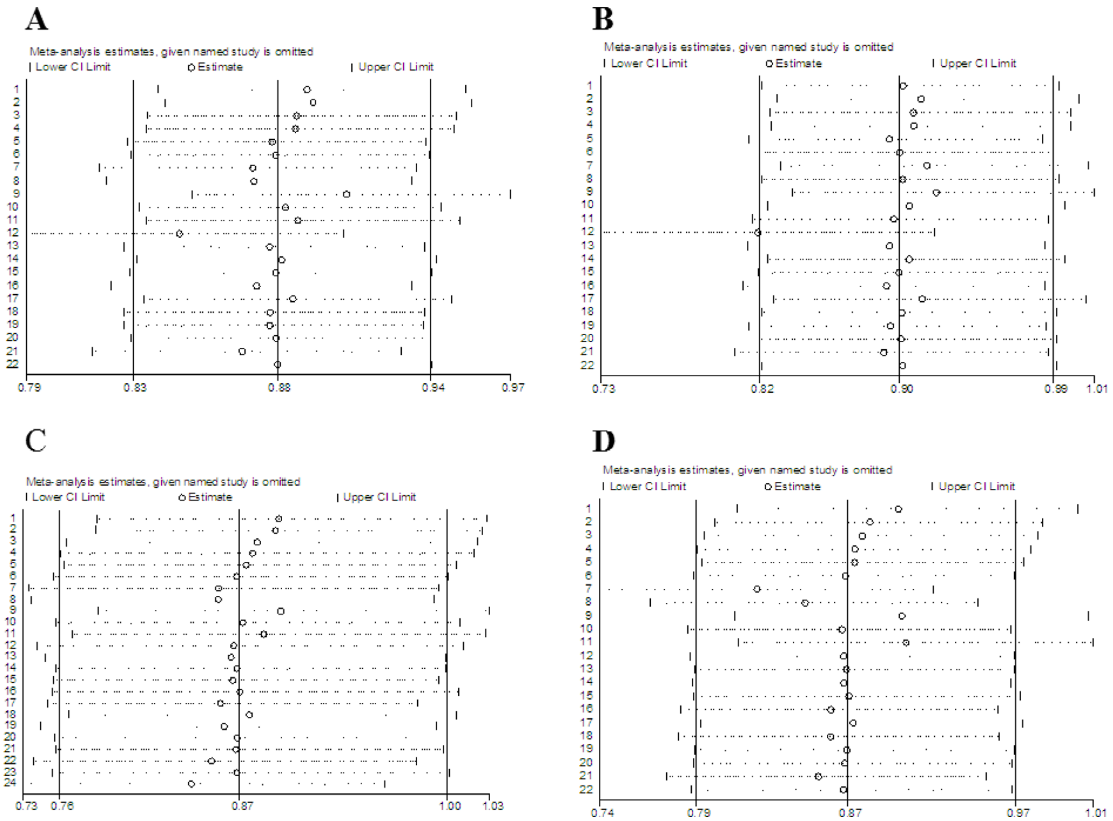

Supplementary Figure 2: Forest plots of sensitivity analysis for the meta-analysisn of rs1800796 and risk of cancer. (**A**) allelic (C vs. G). (**B**) Dominant (CC+ CG vs. GG). (**C**) Recessive (CC vs. CG+GG). (**D**) Additive (CC vs. CG).

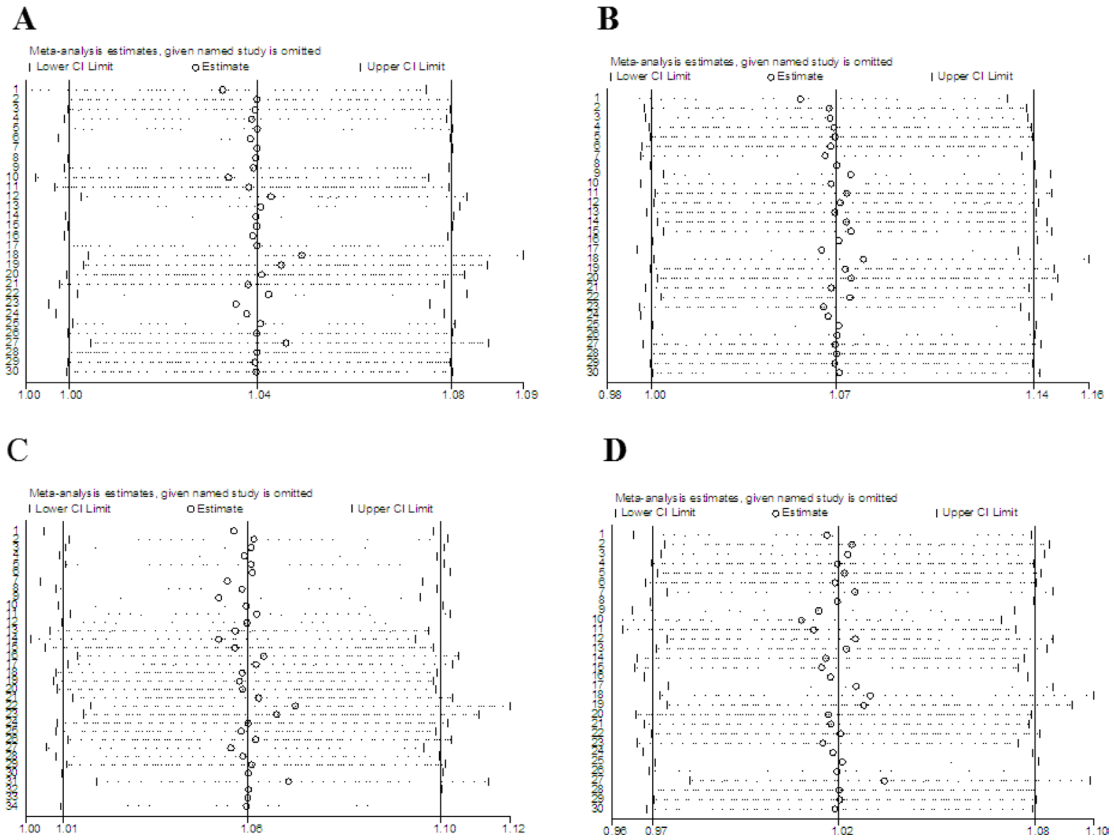

Supplementary Figure 3: :Forest plots of sensitivity analysis for the meta-analysisn of rs1800797 and risk of cancer. (**A**) allelic (G vs. A). (**B**) Dominant (GG+ GA vs. AA). (**C**) Recessive (GG vs. GA+AA). (**D**) Additive (GG vs. GA).

Supplementary Table 1: Characteristics of studies of rs1800795 included in this meta-analysis. See_Supplementary_Table 1

Supplementary Table 2: Characteristics of studies of rs1800796 included in this meta-analysis

Author	Year	Mean Age Case/Control	Cancer type	Country	Sample size Case/Control	Polymorphism
Bai	2013	58/60	lung cancer	China	193/211	rs1800796
Bao	2008	68.2/62.3	Prostate cancer	China	209/209	rs1800796
Chen	2015	64.6/65.2	Prostate cancer	China	212/236	rs1800795<br>rs1800796
Huang	2016	NA	Prostate cancer	China	236/256	rs1800796
Hwang	2003	NA	Gastric cancer	Colombian AND Asian	30/60	rs1800795<br>rs1800796<br>rs1800797
Chen	2015	57.6/58.3	lung cancer	China	622/614	rs1800796
Kang	2007	58.0/58.0	Gastric cancer	Korea	335/335	rs1800796
Kiyohara	2014	68/58	Lung cancer	Japan	462/379	rs1800796
Liang	2012	NA	lung cancer	China	138/138	rs1800796
Lim	2011	63.0/63.6	lung cancer	Singapore	433/1375	rs1800796
Slattery	2008	NA	Breast cancer	Caucasian	575/726	rs1800796
Slattery	2008	NA	Colorectal cancer	Caucasian	2373/2982	rs1800796
Pierce	2009	73.4	prostate cancer	European Americans	175/1934	rs1800796
Pierce	2009	72.7	prostate cancer	European Americans	40/300	rs1800796<br>rs1800795
Seow	2006	62.0/63.4	lung cancer	china	126/162	rs1800796
Smallwood	2008	73.3/72.3	Abdominal Aortic Aneurysm	Australia	677/656	rs1800795<br>rs1800796<br>rs1800797
Sun	2004	NA	prostate cancer	Sweden	1444/866	rs1800796
Tang	2014	48.9/43.6	hepatocellular carcinoma	China	505/395	rs1800796
Tsilidis	2009	62.8/62.8	Colorectal cancer	Caucasian	208/381	rs1800795<br>rs1800796<br>rs1800797
Wang	2011	NA	Prostate Cancer	USA	253/280	rs1800796
Shi WJ	2014	54.9/55.2	Cervical cancer	China	518/518	rs1800795<br>rs1800796
Birmann BM	2009	62/65	multiple myeloma	USA	82/164	rs1800796
Chérel	2009	52.6/50.4	Breast Cancer	France	293/112	rs1800795<br>rs1800796<br>rs1800797

Supplementary Table 3: Characteristics of studies of rs1800797 included in this meta-analysis

Author	Year	Mean Age Case/ Control	Cancer type	Country	Sample size Case/Control	Polymorphism
Castro	2009	48.0/49.6	Cervical cancer	Swedish	973/1763	rs1800797
Gu	2014	NA	Non-Hodgkin Lymphoma	China	157/435	rs1800795 rs1800797
Kane	2015	NA	Non-Hodgkin Lymphoma	American/ European studie	488/1591	rs1800795 rs1800797
Smallwood	2008	73.3/72.3	Abdominal Aortic Aneurysm	Australia	677/656	rs1800795 rs1800796 rs1800797
Zidi	2016	52.0/52.2	Cervical Cancer	Tunisia	112/164	rs1800795 rs1800797
Vasku A	2009	68.0/68.1	Colorectal cancer	Czech	102/101	rs1800795 rs1800797
Kamangar F	2006	58.5/59	Gastric cancer	Finland	256/256	rs1800795 rs1800797
Wang S	2006	NA	Non-Hodgkin Lymphoma	Caucasian	1172/982	rs1800795 rs1800797
Vasku	2004	62/60	Cutaneous T-cell lymphoma	Czech	63/103	rs1800795 rs1800797
Rothman	2006	NA	Non-Hodgkin lymphoma	Europe and North America	3568/4018	rs1800795 rs1800797
Martino	2012	61.6/58.8	Multiple myeloma	Italy		rs1800797
Lan Q	2006	NA	Non-Hodgkin lymphoma	USA	832/601	rs1800795 rs1800797
Ennas	2008	61.8/57.9	Chronic lymphocytic leukaemia	Italy	40/113	rs1800795 rs1800797
Chérel	2009	52.6/50.4	Breast Cancer	France	293/112	rs1800795 rs1800796 rs1800797 (HR)
Snoussi	2005	50/46	Breast Cancer	Tunisia	305/305	rs1800795 rs1800797
Schonfeld	2010	NA	Breast cancer	US	859/1083	rs1800795 rs1800797
Hwang	2003	NA	Gastric cancer	Colombian AND Asian	30/60	rs1800795 rs1800796 rs1800797
Tsilidis	2009	62.8/62.8	Colorectal cancer	Caucasian	208/381	rs1800795 rs1800796 rs1800797

Supplementary Table 4: Genotype frequencies of rs1800795 included in this meta-analysis. See_ Supplementary_Table 4

Supplementary Table 5: Genotype frequencies of rs1800796 included in this meta-analysis

Author	Case			Control			MAF		HWE
	CC	CG	GG	CC	CG	GG	Case	Control	
Bai	86	89	18	125	69	16	0.68	0.76	0.15
Bao	50	39	47	65	27	28	0.51	0.63	0.16
Chen	79	96	31	110	102	25	0.62	0.71	0.85
Huang	117	88	31	144	89	23	0.68	0.73	0.09
Hwang(C)	3	16	11	5	7	18	0.37	0.57	0.33
Hwang(A)	16	13	1	16	13	1	0.90	0.90	0.23
Chen	349	229	44	309	252	53	0.64	0.58	0.87
Kang	178	133	21	169	140	17	0.67	0.59	0.08
Kiyohara	259	175	28	250	116	3	0.68	0.65	0.09
Liang	100	29	9	105	30	3	0.83	0.85	0.62
Lim	163	123	12	449	231	38	0.75	0.83	0.25
Slattery	242	333		320	406				
Slattery	153	1022		141	1188				
Slattery	27	311	2035	34	366	2582	0.12	0.15	0.07
Pierce(C)	0	19	156	2	192	1740	0.11	0.16	0.16
Pierce(A)	1	2	37	2	46	253	0.07	0.08	0.47
Seow	70	46	8	97	55	10	0.75	0.78	
Smallwood	10	64	587	3	70	582	0.65	0.64	0.57
Sun	2	109	1226	4	74	675	0.07	0.09	0.21
Tang	78	34	7	64	35	6	0.80	0.75	0.68
Tsilidis	2	19	180	3	30	329	0.09	0.11	0.19
Wang	1	19	233	0	25	255	0.06	0.09	0.43
Shi WJ	50	167	301	27	181	310	0.26	0.15	0.93
Birmann BM	67	8	1	141	18	0	0.91	0.94	0.45

Supplementary Table 6: Genotype frequencies of rs1800797 included in this meta-analysis. See_ Supplementary_Table 6
