# Supplementary material for: Genetic polymorphisms of IL-6 promoter in cancer susceptibility and prognosis: a meta-analysis
Source: Oncotarget. 2018 Jan 5;9(15):12351–64. doi: 10.18632/oncotarget.24033 (PMC5844752; doi:10.18632/oncotarget.24033)
Supplement: Supplementary file 2 [file oncotarget-09-12351-s002.docx]

**Supplementary table 1: Characteristics of studies of rs1800795 included in this meta-analysis**

| **Author** | **Year** | **Mean Age**  **Case/Control** | **Cancer type** | **Country** | **Sample size** | **Polymorphism** |
| --- | --- | --- | --- | --- | --- | --- |
|  |  |  |  |  | **Case/****Control** |  |
| Lima Júnior | 2016 | 33.9/37.2 | Cervical cancer | Brazil | 108/108 | rs1800795 |
| Nogueira | 2006 | 50.0/53.0 | Cervical cancer | Brazil | 56/253 | rs1800795 |
| Grimm | 2011 | 34.6/31.1 | Cervical cancer | Austria | 131/209 | rs1800795 |
| Shi WJ | 2014 | 54.9/55.2 | Cervical cancer | China | 518/518 | rs1800795  rs1800796 |
| Vasku A | 2009 | 68.0/68.1 | Sporadic colorectal cancer | Czech | 102/101 | rs1800795  rs1800797 |
| Cacev T | 2010 | 64.5/63.1 | Sporadic Colon Cancer | Croatian | 160/160 | rs1800795 |
| Gangwar | 2009 | 45.0/46.0 | Cervical cancer | India | 160/200 | rs1800795 |
| Lima Júnior | 2012 | NA | Cervical cancer | Brazil | 345/345 | rs1800795 |
| Landi S | 2003 | NA | colorectal cancer | Spain | 377/326 | rs1800795 |
| Marc J | 2006 | 60.0/57.0 | colorectal cancer | USA | 244/231 | rs1800795 |
| George T | 2006 | 64.2/62.6 | colorectal cancer | Greek | 222/200 | rs1800795 |
| Slattery | 2007 | NA | Colon and rectal cancer | USA | 2380/2990 | rs1800795  rs1800796 |
| Vogel | 2007 | 59.0/56.0 | colorectal cancer | Denmark | 355/753 | rs1800795 |
| Küry S | 2008 | 64.2/61.5 | sporadic colorectal cancer | French | 1023/1121 | rs1800795 |
| Wilkening S | 2008 | 56.8/56.8 | colorectal cancer | Swedish | 308/585 | rs1800795 |
| Tsilidis KK | 2009 | 62.8/62.8 | colorectal cancer | USA | 208/381 | rs1800795  rs1800796 |
| Ognjanovic S | 2010 | 62.5/62.0 | Colorectal adenom | USA | 271/539 | rs1800795 |
| Crusius | 2008 | NA | gastric cancer | European | 439/1138 | rs1800795 |
| El-omar | 2003 | 66.0/66.0 | Esophageal cancer | USA | 53/210 | rs1800795 |
| El-omar | 2003 | 65.0/66.0 | Esophageal cancer | USA | 108/210 | rs1800795 |
| El-omar | 2003 | 66.0/66.0 | gastric cancer | USA | 126/210 | rs1800795 |
| El-omar | 2003 | 70.0/66.0 | gastric cancer | USA | 188/210 | rs1800795 |
| Kamangar F | 2006 | 58.5/59 | gastric cancer | Finland | 256/256 | rs1800795  rs1800797 |
| Chris D | 2006 | 71.0/39.2 | gastric cancer | British Caucasian | 203/266 | rs1800795 |
| Zheng C | 2000 | 67.0/NA | Multiple myeloma | Sweden | 73/129 | rs1800795 |
| Wang S | 2006 | NA | Non-Hodgkin Lymphoma | Caucasian | 1172/982 | rs1800795  rs1800797 |
| Vasku | 2004 | 62/60 | cutaneous T-cell lymphoma | Czech | 63/103 | rs1800795  rs1800797 |
| Rothman | 2006 | NA | non-Hodgkin lymphoma | Europe and North America | 3568/4018 | rs1800795  rs1800797 |
| Rausz E | 2013 | 42.9/68.0 | Mastocytosis | Europe | 66/99 | rs1800795 |
| Mazur | 2005 | 62/NA | multiple myeloma | Poland | 54/50 | rs1800795 |
| Lan Q | 2006 | NA | non-Hodgkin lymphoma | USA | 832/601 | rs1800795  rs1800797 |
| Hulkkonen J | 2000 | NA | non-Hodgkin lymphoma | Finland | 36/400 | rs1800795 |
| Hohaus | 2007 |  | Hodgkin’s lymphoma |  |  | rs1800795  HR |
| Ennas | 2008 | 61.8/57.9 | chronic lymphocytic leukaemia | Italy | 40/113 | rs1800795  rs1800797 |
| Chakraborty | 2014 | 56.0/52.5 | multiple myeloma | India | 103/117 | rs1800795 |
| Duch CR | 2007 | NA | multiple myeloma | Brazil | 52/60 | rs1800795 |
| Cozen | 2004 | NA | Hodgkin lymphomaHD young adult | USA | 88/87 | rs1800795 |
| Andrie | 2009 | childhood | HD childhood | Greece | 37/48 | rs1800795 |
| Aladzsity | 2009 | 65/68 | myelodysplasia and multiple myeloma | Hungary | 100/99 | rs1800795 |
| Madeleine | 2011 | NA | **breast cancer** |  |  | rs1800795 |
| Joshi | 2014 | 40.0/32.0 | **breast cancer** | India | 182/236 | rs1800795 |
| Dossus L | 2010 | 63.1/63.1 | Breast Cancer | USA and Europe | 6292/8135 | rs1800795 |
| Dossus L | 2010 | 63.1/63.1 | prostate cancer |  | 8008/8604 | rs1800795 |
| Chérel | 2009 | 52.6/50.4 | Breast Cancer | France | 293/112 | rs1800795  rs1800796  rs1800797  (HR) |
| Slattery M | 2008 | NA | Breast Cancer | Hispanic/American Indian |  | rs1800795 |
| Slattery | 2008 | NA | Breast Cancer |  |  |  |
| Vogel | 2006 | NA | Breast Cancer | Denmark | 361/361 | rs1800795 |
| González-Zuloeta | 2006 | 67.8/70.8 | Breast Cancer | Netherlands | 171/3651 | rs1800795 |
| Balasubramanian | 2006 | 57/63 | Breast Cancer | UK | 497/490 | rs1800795 |
| Snoussi | 2005 | 50/46 | Breast Cancer | Tunisia | 305/305 | rs1800795  rs1800797 |
| Hefler | 2005 | 54.9/53.3 | Breast Cancer | Austria | 228/228 | rs1800795 |
| Smith | 2004 | 59.6/40.3 | Breast Cancer | UK | 144/263 | rs1800795 |
| Litovkin | 2007 |  |  | Ukraine |  | rs1800795 |
| Brenner | 2007 | 51/49 | adult glioma | USA | 431/611 | rs1800795 |
| Brenner | 2007 | 47/59 | adult glioma | USA | 325/579 | rs1800795 |
| Smallwood | 2008 | 73.3/72.3 | Abdominal Aortic Aneurysm | Australia | 677/656 | rs1800795  rs1800796  rs1800797 |
| Slattery ML | 2009 | NA | colon cancer | USA | 1039/2014 | rs1800795 |
| Pierce B L | 2009 | 73.4/73.3 | Prostate cancer | European Americans | 1923/2234 | rs1800795  rs1800796  **HR** |
| Pierce B L | 2009 | 72.7/73.3 | Prostate cancer | African Americans | 300/2234 | rs1800795  rs1800796  **HR** |
| Abulí A | 2011 | NA | colorectal cancer | Spain | 515/515 | rs1800795 |
| Pooja | 2012 | 44.9/39.0 | Breast Cancer | India | 200/200 | rs1800795 |
| Pohjanen | 2013 | 65.9/53.8 | Gastric cancer | Finland | 61/179 | rs1800795 |
| Totaro F | 2013 | NA | Neuroblastoma | Italian | 326/511 | rs1800795  HR |
| Mandal | 2014 | 63.7/60.2 | prostate cancer | USA | 164/140 | rs1800795 |
| Oduor | 2014 | 5/7 | Burkitt Lymphoma | Africa | 117/88 | rs1800795 |
| Gu X | 2014 | NA | Non-Hodgkin Lymphoma | China | 157/204 | rs1800795  rs1800797 |
| Talaat | 2015 | NA | Diffuse Large B-Cell Lymphoma | Egypt | 100/119 | rs1800795 |
| Chen | 2016 | 64.5/65.2 | prostate cancer | China | 212/236 | rs1800795  rs1800796 |
| Matsusaka |  |  |  |  |  | HR |
| Zidi | 2016 | 52.0/52.2 | Cervical Cancer | Tunisia | 112/164 | rs1800795  rs1800797 |
| Pu | 2016 | 54.8/56.5 | Cervical Cance | China | 360/728 | rs1800795 |
| Özgen | 2009 | 43.1/43.8 | papillary thyroid carcinoma | Turkish | 42/340 | rs1800795 |
| Schonfeld | 2010 | NA | Breast cancer | US | 859/1083 | rs1800795  rs1800797 |
| ahirwar | 2008 | 61.6/58.3 | bladder cancer | India | 136/200 | rs1800795 |
| Basturk | 2005 | NA | Kidney cancer | Turkey | 29/50 | rs1800795 |
| berkivic | 2007 | NA | Gastric cancer | Caucasian | 80/162 | rs1800795 |
| campa | 2004 | NA | Lung Cancer | Europe | 2315/2115 | rs1800795 |
| cordano | 2005 | NA | Hodgkin’s lymphoma | Caucasian | 408/349 | rs1800795 |
| Seifart | 2005 | NA | lung cancer | Caucasian | 365/243 | rs1800795 |
| gaur | 2011 | 51.4/NA | oral cancer | India | 140/120 | rs1800795 |
| kesarwani | 2008 | 62.5/59.5 | Prostate Cancer | India | 200/200 | rs1800795 |
| malaponte | 2013 | 63/61 | Cancer | Italy | 320/215 | rs1800795 |
| michaud | 2006 | 67.1/66.6 | Prostate Cancer | USA | 1526/1842 | rs1800795 |
| ognjanovic | 2009 | 60.5/59.5 | lung cancer | USA | 120/230 | rs1800795 |
| vairaktaris | 2008 |  | Oral cancer | Caucasians. | 162/168 | rs1800795 |
| vogel | 2008 | NA | lung cancer | *Denmark* | 403/744 | rs1800795 |
| bushley | 2004 | NA | ovarian cancer | USA | 182/219 | rs1800795 |
| Hwang | 2003 | NA | Gastric cancer | Colombian  AND Asian | 30/60 | rs1800795  rs1800796  rs1800797 |
| Kane | 2015 | NA | Non-Hodgkin Lymphoma | American/  European | 488/1591 | rs1800795  rs1800797 |
