# Supplementary material for: Genetic polymorphisms of IL-6 promoter in cancer susceptibility and prognosis: a meta-analysis
Source: Oncotarget. 2018 Jan 5;9(15):12351–64. doi: 10.18632/oncotarget.24033 (PMC5844752; doi:10.18632/oncotarget.24033)
Supplement: Supplementary file 3 [file oncotarget-09-12351-s003.docx]

**Supplementary table 4. Genotype frequencies of rs1800795 included in this meta-analysis**

| **Author** | **Case** | | | **Control** | | | **MAF** | | **HWE** |
| --- | --- | --- | --- | --- | --- | --- | --- | --- | --- |
|  | **GG** | **GC** | **CC** | **GG** | **GC** | **CC** | **Case** | **Control** |  |
| Lima Júnior | 67 | 38 | 3 | 64 | 35 | 9 | 0.79 | 0.76 | 0.19 |
| Nogueira | 24 | 32 | 0 | 148 | 120 | 3 | 0.63 | 0.75 | 0.17 |
| Grimm | 55 | 51 | 25 | 85 | 96 | 27 | 0.62 | 0.64 | 0.48 |
| Shi WJ | 160 | 253 | 105 | 181 | 259 | 78 | 0.55 | 0.60 | 0.35 |
| Vasku A | 32 | 46 | 22 | 31 | 47 | 22 | 0.55 | 0.55 | 0.60 |
| Cacev T | 64 | 70 | 26 | 68 | 75 | 17 | 0.62 | 0.66 | 0.58 |
| Gangwar | 107 | 36 | 17 | 142 | 51 | 7 | 0.78 | 0.84 | 0.37 |
| Lima Júnior | 72 | 39 | 4 | 67 | 37 | 11 | 0.80 | 0.74 | 0.09 |
| Landi S | 145 | 133 | 33 | 133 | 180 | 48 | 0.68 | 0.62 | 0.29 |
| Marc J | 79 | 90 | 35 | 83 | 81 | 26 | 0.61 | 0.65 | 0.38 |
| George T | 111 | 76 | 35 | 64 | 86 | 50 | 0.67 | 0.54 | 0.06 |
| Slattery(C) | 631 | 696 | 246 | 728 | 897 | 347 | 0.62 | 0.60 | 0.14 |
| Slattery(R) | 321 | 347 | 109 | 411 | 438 | 146 | 0.64 | 0.63 | 0.09 |
| Vogel | 98 | 168 | 89 | 204 | 364 | 185 | 0.51 | 0.51 | 0.37 |
| Küry S | 363 | 489 | 171 | 435 | 504 | 182 | 0.59 | 0.61 | 0.08 |
| Wilkening S | 79 | 163 | 61 | 162 | 297 | 121 | 0.53 | 0.54 | 0.48 |
| Tsilidis KK | 68 | 93 | 39 | 113 | 170 | 71 | 0.58 | 0.56 | 0.63 |
| Ognjanovic S | 173 | 74 | 22 | 357 | 136 | 43 | 0.54 | 0.61 | 0.17 |
| Crusius | 140 | 224 | 75 | 415 | 517 | 206 | 0.57 | 0.59 | 0.06 |
| El-omar(S) | 13 | 6 | 5 | 83 | 98 | 28 | 0.67 | 0.63 | 0.91 |
| El-omar(A) | 20 | 33 | 13 | 83 | 98 | 28 | 0.55 | 0.63 | 0.91 |
| El-omar(C) | 22 | 31 | 5 | 83 | 98 | 28 | 0.65 | 0.63 | 0.91 |
| El-omar(N) | 33 | 21 | 11 | 83 | 98 | 28 | 0.67 | 0.63 | 0.91 |
| Kamangar F | 21 | 54 | 27 | 51 | 58 | 43 | 0.41 | 0.48 | 0.23 |
| Chris D | 71 | 83 | 43 | 79 | 101 | 44 | 0.57 | 0.58 | 0.26 |
| Zheng C(MM) | 22 | 36 | 15 | 33 | 69 | 26 | 0.55 | 0.53 | 0.36 |
| Zheng C(PCD) | 29 | 49 | 22 | 33 | 69 | 26 | 0.54 | 0.53 | 0.36 |
| Wang S(N) | 329 | 436 | 158 | 224 | 352 | 127 | 0.59 | 0.57 | 0.58 |
| Wang S(B) | 397 | 379 | 143 | 393 | 410 | 138 | 0.64 | 0.64 | 0.07 |
| Wang S(T) | 23 | 39 | 11 | 393 | 410 | 138 | 0.58 | 0.64 | 0.07 |
| Wang S(D) | 143 | 161 | 51 | 393 | 410 | 138 | 0.63 | 0.64 | 0.07 |
| Wang S(F) | 116 | 107 | 47 | 393 | 410 | 138 | 0.63 | 0.64 | 0.07 |
| Wang S(S) | 63 | 55 | 26 | 393 | 410 | 138 | 0.63 | 0.64 | 0.07 |
| Wang S(M) | 46 | 32 | 14 | 393 | 410 | 138 | 0.67 | 0.64 | 0.07 |
| Vasku | 19 | 35 | 9 | 36 | 46 | 23 | 0.58 | 0.56 | 0.26 |
| Rothman(N) | 1097 | 1470 | 499 | 1277 | 1658 | 564 | 0.60 | 0.60 | 0.51 |
| Rothman(D) | 419 | 527 | 217 | 1427 | 1858 | 664 | 0.59 | 0.60 | 0.16 |
| Rothman(F) | 313 | 417 | 163 | 1427 | 1858 | 664 | 0.58 | 0.60 | 0.16 |
| Rausz E | 18 | 32 | 16 | 36 | 49 | 14 | 0.52 | 0.61 | 0.68 |
| Mazur | 11 | 31 | 12 | 16 | 28 | 6 | 0.49 | 0.60 | 0.24 |
| Lan Q(N) | 211 | 231 | 68 | 241 | 264 | 85 | 0.64 | 0.63 | 0.36 |
| Lan Q(B) | 160 | 182 | 64 | 241 | 264 | 85 | 0.62 | 0.63 | 0.36 |
| Lan Q(T) | 23 | 14 | 1 | 241 | 264 | 85 | 0.79 | 0.63 | 0.36 |
| Hulkkonen J | 14 | 13 | 8 | 118 | 201 | 81 | 0.59 | 0.55 | 0.78 |
| Ennas | 17 | 16 | 6 | 64 | 43 | 5 | 0.64 | 0.76 | 0.51 |
| Chakraborty | 54 | 40 | 9 | 68 | 38 | 11 | 0.72 | 0.74 | 0.11 |
| Duch CR | 28 | 22 | 2 | 35 | 23 | 2 | 0.75 | 0.78 | 0.44 |
| Cozen | 41 | 37 | 8 | 25 | 39 | 14 | 0.69 | 0.57 | 0.86 |
| Andrie(H) | 23 | 11 | 2 | 47 | 30 | 4 | 0.79 | 0.77 | 0.78 |
| Andrie(N) | 32 | 12 | 1 | 47 | 30 | 4 | 0.84 | 0.77 | 0.78 |
| Aladzsity(MDS) | 35 | 56 | 11 | 36 | 49 | 14 | 0.61 | 0.61 | 0.68 |
| Aladzsity(MM) | 37 | 43 | 17 | 36 | 49 | 14 | 0.60 | 0.61 | 0.68 |
| Joshi | 148 | 28 | 2 | 166 | 63 | 3 | 0.91 | 0.85 | 0.27 |
| Dossus L(B) | 2847 | 2523 | 820 | 3707 | 3324 | 1035 | 0.66 | 0.67 | 0.06 |
| Dossus L(P) | 3594 | 3218 | 1125 | 3832 | 3402 | 1274 | 0.66 | 0.65 | 0.09 |
| Chérel | 102 | 131 | 60 | 29 | 58 | 25 | 0.57 | 0.52 | 0.70 |
| Slattery M | 80 | | 8 | 95 | | 7 |  |  |  |
| Vogel | 108 | 167 | 86 | 98 | 177 | 86 | 0.53 | 0.52 | 0.73 |
| González-Zuloeta | 55 | 86 | 30 | 1286 | 1733 | 632 | 0.57 | 0.59 | 0.25 |
| Balasubramanian | 170 | 244 | 83 | 168 | 235 | 87 | 0.59 | 0.58 | 0.76 |
| Snoussi | 199 | 98 | 8 | 150 | 46 | 4 | 0.81 | 0.87 | 0.83 |
| Hefler | 78 | 139 | 52 | 91 | 105 | 31 | 0.55 | 0.63 | 0.94 |
| Smith | 57 | 67 | 20 | 79 | 101 | 44 | 0.63 | 0.58 | 0.26 |
| Litovkin | 26 | 39 | 8 | 30 | 39 | 9 | 0.63 | 0.64 | 0.49 |
| Brenner(N) | 129 | 68 | 50 | 162 | 238 | 88 | 0.66 | 0.58 | 0.97 |
| Brenner(NO) | 93 | 164 | 50 | 157 | 265 | 123 | 0.58 | 0.53 | 0.58 |
| Smallwood | 222 | 300 | 104 | 224 | 302 | 124 | 0.59 | 0.58 | 0.22 |
| Slattery ML | 430 | 741 | | 409 | 920 | |  |  |  |
| Slattery ML | 389 | 186 | | 460 | 266 | |  |  |  |
| Slattery ML | 631 | 696 | 246 | 728 | 897 | 347 | 0.62 | 0.60 | 0.15 |
| Pierce B L(E) | 48 | 96 | 31 | 696 | 901 | 336 | 0.55 | 0.59 | 0.13 |
| Pierce B L(A) | 34 | 5 | 1 | 250 | 48 | 2 | 0.91 | 0.91 | 0.85 |
| Abulí A | 586 | 635 | 184 | 593 | 623 | 172 | 0.64 | 0.65 | 0.67 |
| Pooja | 80 | 120 | | 52 | 148 | |  |  |  |
| Pohjanen(D) | 8 | 15 | 2 | 37 | 86 | 56 | 0.62 | 0.45 | 0.71 |
| Pohjanen(N) | 6 | 19 | 6 | 37 | 86 | 56 | 0.50 | 0.45 | 0.71 |
| Totaro F | 176 | 125 | 25 | 295 | 184 | 32 | 0.73 | 0.76 | 0.65 |
| Mandal(A) | 58 | 16 | 6 | 48 | 14 | 0 | 0.83 | 0.89 | 0.06 |
| Mandal(C) | 50 | 28 | 6 | 26 | 30 | 22 | 0.76 | 0.53 | 0.32 |
| Oduor | 113 | 4 | 0 | 88 | 0 | 0 | 0.98 | 1 | 0.04 |
| Gu X(N) | 83 | 10 | 0 | 198 | 6 | 0 | 0.95 | 0.99 | 0.83 |
| Gu X(B) | 71 | 7 | 0 | 198 | 6 | 0 | 0.96 | 0.99 | 0.83 |
| Gu X(D) | 34 | 0 | 0 | 198 | 6 | 0 | 1 | 0.99 | 0.83 |
| Gu X(M) | 18 | 3 | 0 | 198 | 6 | 0 | 0.93 | 0.99 | 0.83 |
| Talaat | 86 | 14 | 0 | 92 | 26 | 1 | 0.93 | 0.88 | 0.57 |
| Chen | 131 | 64 | 17 | 158 | 67 | 11 | 0.77 | 0.81 | 0.27 |
| zidi | 81 | 25 | 6 | 133 | 25 | 6 | 0.83 | 0.89 | 0.07 |
| pu | 185 | 141 | 34 | 476 | 220 | 32 | 0.71 | 0.80 | 0.31 |
| Özgen | 21 | 14 | 7 | 143 | 171 | 26 | 0.67 | 0.67 | 0.13 |
| Schonfeld | 274 | 408 | 156 | 379 | 487 | 211 | 0.57 | 0.58 | 0.15 |
| Gunter | 79 | 90 | 35 | 83 | 81 | 26 | 0.61 | 0.65 | 0.38 |
| ahirwar | 86 | 24 | 26 | 130 | 56 | 14 | 0.72 | 0.79 | 0.27 |
| Basturk | 15 | 10 | 0 | 27 | 13 | 9 | 0.80 | 0.68 | 0.06 |
| berkivic | 25 | 44 | 11 | 69 | 75 | 18 | 0.59 | 0.66 | 0.72 |
| campa | 629 | 954 | 412 | 615 | 993 | 374 | 0.55 | 0.56 | 0.45 |
| Cordano(H) | 134 | 197 | 77 | 106 | 184 | 59 | 0.57 | 0.57 | 0.17 |
| Cordano(N) | 18 | 16 | 7 | 106 | 184 | 59 | 0.63 | 0.57 | 0.17 |
| Cordano(C) | 115 | 181 | 70 | 106 | 184 | 59 | 0.56 | 0.57 | 0.17 |
| Seifart(SCLC) | 19 | 16 | 4 | 14 | 19 | 7 | 0.69 | 0.59 | 0.90 |
| Seifart(NSCLC) | 28 | 36 | 13 | 29 | 41 | 5 | 0.60 | 0.66 | 0.06 |
| Seifart(SCC) | 17 | 19 | 4 | 17 | 18 | 4 | 0.66 | 0.67 | 0.81 |
| Seifart(AC) | 10 | 11 | 5 | 8 | 17 | 0 | 0.60 | 0.66 | 0.13 |
| gaur | 98 | 35 | 7 | 65 | 41 | 14 | 0.83 | 0.71 | 0.07 |
| kesarwani | 102 | 84 | 14 | 103 | 87 | 10 | 0.72 | 0.73 | 0.12 |
| malaponte | 141 | 179 | | 79 | 136 | |  |  |  |
| michaud | 170 | 223 | 91 | 230 | 293 | 90 | 0.58 | 0.61 | 0.83 |
| ognjanovic | 71 | 46 | | 103 | 118 | |  |  |  |
| vairaktaris | 102 | 42 | 18 | 60 | 90 | 6 | 0.76 | 0.67 | 0.07 |
| vogel | 105 | 202 | 96 | 204 | 361 | 179 | 0.51 | 0.52 | 0.44 |
| Theodoropoulos | 111 | 76 | 35 | 64 | 86 | 50 | 0.67 | 0.54 | 0.06 |
| bushley | 143 | 34 | 5 | 163 | 46 | 9 | 0.88 | 0.85 | 0.19 |
| Snoussi | 199 | 98 | 8 | 150 | 46 | 4 | 0.81 | 0.87 | 0.83 |
| Hwang(C) | 19 | 9 | 2 | 41 | 17 | 2 | 0.78 | 0.83 | 0.88 |
| Hwang(A) | 30 | 0 | 0 | 60 | 0 | 0 |  |  |  |
| Kane(D) | 271 | 495 | | 703 | 1362 | |  |  |  |
| Kane(F) | 222 | 415 | | 703 | 1362 | |  |  |  |
| Kane(C) | 81 | 151 | | 541 | 1056 | |  |  |  |
| Kane(L) | 777 | 1408 | | 703 | 1362 | |  |  |  |
