# Supplementary material for: Genetic polymorphisms of IL-6 promoter in cancer susceptibility and prognosis: a meta-analysis
Source: Oncotarget. 2018 Jan 5;9(15):12351–64. doi: 10.18632/oncotarget.24033 (PMC5844752; doi:10.18632/oncotarget.24033)
Supplement: Supplementary file 4 [file oncotarget-09-12351-s004.docx]

**Supplementary table 6. Genotype frequencies of rs1800797 included in this meta-analysis**

| **Author** | **Case** | | | **Control** | | | **MAF** | | **HWE** |
| --- | --- | --- | --- | --- | --- | --- | --- | --- | --- |
|  | **GG** | **GA** | **AA** | **GG** | **GA** | **AA** | **Case** | **Control** |  |
| Castro | 301 | 473 | 180 | 504 | 841 | 370 | 0.56 | 0.63 | 0.58 |
| Gu(N) | 83 | 10 | 0 | 198 | 6 | 0 | 0.95 | 0.98 | 0.83 |
| Gu(B) | 71 | 7 | 0 | 198 | 6 | 0 | 0.96 | 0.98 | 0.83 |
| Gu(D) | 34 | 0 | 0 | 198 | 6 | 0 | 1.00 | 0.98 | 0.83 |
| Gu(M) | 18 | 3 | 0 | 198 | 6 | 0 | 0.93 | 0.98 | 0.83 |
| Kane(D) | 169 | 315 | | 536 | 1031 | |  |  |  |
| Kane(F) | 160 | 258 | | 536 | 1031 | |  |  |  |
| Kane(C) | 85 | 146 | | 536 | 1031 | |  |  |  |
| Kane(L) | 615 | 1048 | | 536 | 1031 | |  |  |  |
| Smallwood | 245 | 312 | 108 | 236 | 306 | 109 | 0.60 | 0.62 | 0.56 |
| Zidi | 74 | 34 | 4 | 116 | 33 | 15 | 0.81 | 0.86 | 0.07 |
| Vasku A | 31 | 46 | 23 | 33 | 47 | 20 | 0.53 | 0.56 | 0.66 |
| Kamangar F | 61 | 86 | 56 | 25 | 59 | 26 | 0.56 | 0.43 | 0.45 |
| Wang(N) | 325 | 442 | 167 | 228 | 361 | 128 | 0.58 | 0.51 | 0.47 |
| Wang(B) | 396 | 379 | 133 | 379 | 408 | 137 | 0.64 | 0.63 | 0.12 |
| Wang(T) | 22 | 37 | 11 | 379 | 408 | 137 | 0.57 | 0.63 | 0.12 |
| Wang(D) | 145 | 160 | 49 | 379 | 408 | 137 | 0.51 | 0.63 | 0.12 |
| Wang(F) | 116 | 109 | 41 | 379 | 408 | 137 | 0.67 | 0.63 | 0.12 |
| Wang(S) | 65 | 53 | 25 | 379 | 408 | 137 | 0.69 | 0.63 | 0.12 |
| Wang(M) | 40 | 35 | 13 | 379 | 408 | 137 | 0.62 | 0.63 | 0.12 |
| Vasku | 15 | 39 | 7 | 37 | 41 | 25 | 0.62 | 0.56 | 0.05 |
| Rothman(N) | 998 | 1243 | 417 | 1151 | 1423 | 494 | 0.58 | 0.61 | 0.12 |
| Rothman(D) | 300 | 386 | 127 | 1151 | 1423 | 494 | 0.61 | 0.61 | 0.12 |
| Rothman(F) | 232 | 270 | 96 | 1151 | 1423 | 494 | 0.58 | 0.61 | 0.12 |
| Martino | 97 | 89 | 15 | 103 | 109 | 22 | 0.70 | 0.67 | 0.37 |
| Lan Q(B) | 159 | 171 | 61 | 233 | 254 | 84 | 0.63 | 0.63 | 0.28 |
| Lan Q(T) | 24 | 12 | 1 | 233 | 254 | 84 | 0.77 | 0.63 | 0.28 |
| Lan Q(N) | 212 | 217 | 64 | 233 | 254 | 84 | 0.68 | 0.63 | 0.28 |
| Ennas | 17 | 15 | 6 | 65 | 42 | 4 | 0.64 | 0.68 | 0.38 |
| Snoussi | 197 | 99 | 9 | 149 | 47 | 4 | 0.81 | 0.86 | 0.90 |
| Schonfeld | 272 | 395 | 151 | 393 | 478 | 199 | 0.61 | 0.59 | 0.18 |
| Hwang(C) | 20 | 8 | 2 | 42 | 16 | 2 | 0.80 | 0.71 | 0.76 |
| Hwang(A) | 30 | 0 | 0 | 60 | 0 | 0 | 1 | 1 |  |
| Tsilidis | 70 | 96 | 37 | 120 | 179 | 68 | 0.58 | 0.57 | 0.93 |
